# Population-based estimates of the global prevalence and carrier frequency of apparent mineralocorticoid excess caused by 11β-hydroxysteroid dehydrogenase type 2 deficiency

**DOI:** 10.1186/s13023-025-04160-x

**Published:** 2025-11-28

**Authors:** Nipith Charoenngam, Chalermkiat Kansuttiviwat, Palinee Chinsawangwatanakul

**Affiliations:** 1 Division of Endocrinology, Massachusetts General Hospital, Harvard Medical School, Boston, MA USA; 2 Department of Medicine, Faculty of Medicine Siriraj Hospital, Mahidol University, 2 Wanglang Road Bangkoknoi, Bangkok, 10700 Thailand; 3 Department of Medicine, University Hospital, Case Western Reserve University, Cleveland, OH USA; 4 Center of Human Genetics, University Hospital, Case Western Reserve University, Cleveland, OH USA; 5 Faculty of Medicine, Prince of Songkla University, Songkla, Thailand

## Abstract

**Background:**

Apparent mineralocorticoid excess (AME) is a rare autosomal recessive disorder caused by biallelic inactivating variants in the HSD11B2 gene resulting in hypertension and electrolyte abnormalities due to cortisol-mediated activation of mineralocorticoid receptors. We aimed to utilize data from a large genomic database to estimate the prevalence and carrier frequency of AME, with stratification by ethnic ancestry to identify potential disparities.

**Methods:**

We analyzed sequencing data from 807,162 unrelated individuals in the gnomAD v4.1 database, representing diverse ethnic ancestries. Potentially disease-causing *HSD11B2* variants were identified using three approaches: classification as pathogenic/likely pathogenic (P/LP) in ClinVar, association with AME based on systematic review of the literature, or predicted deleterious effects by in silico tools. Carrier frequency was calculated based on combined allele frequencies. Disease prevalence was estimated using Hardy-Weinberg equilibrium assumptions. Analyses were stratified by ancestry and variant selection criteria.

**Results:**

Of 1,506 *HSD11B2* variants in gnomAD, 160 qualified as potentially pathogenic. Using strict criteria (ClinVar/literature), the global carrier frequency was 24.5/100,000 individuals and disease prevalence was 0.6/10,000,000. With liberal criteria (including in silico predictions), these increased to 82.6/100,000 and 6.8/10,000,000. The highest carrier frequencies were observed in Middle Eastern individuals (132.0/100,000 under strict criteria) and South Asians (147.1/100,000 under liberal criteria). Several variants were enriched in specific populations.

**Conclusion:**

This study provides the first global, ancestry-stratified estimates of AME carrier frequency and prevalence, revealing variability by ancestry and variant classification. Results highlight the need for improved variant curation and broader population representation in genomic research.

**Supplementary Information:**

The online version contains supplementary material available at 10.1186/s13023-025-04160-x.

## Introduction

Apparent mineralocorticoid excess (AME) is a rare autosomal recessive disorder caused by deficiency of 11β-hydroxysteroid dehydrogenase type 2 (HSD11B2), the enzyme responsible for converting active glucocorticoids such as cortisol and corticosterone to their inactive metabolites, cortisone and 11-deoxycorticosterone, in the kidney [1, 2]. In the absence of adequate HSD11B2 activity, cortisol remains active and aberrantly stimulates mineralocorticoid receptors that are normally specific to aldosterone, resulting in sodium and water retention, potassium wasting and hypertension that mimics primary aldosteronism despite suppressed renin and aldosterone levels [1, 2]. Clinical manifestations typically begin in early childhood and may include severe hypertension with associated end-organ damage, as well as nephrocalcinosis and hypokalemic paralysis in some cases [3]. Early diagnosis and treatment of AME are crucial to prevent end-organ complications. Treatment involves mineralocorticoid receptor blockade, along with exogenous glucocorticoids to suppress endogenous cortisol production [3, 4].

Although over 50 *HSD11B2* variants associated with AME have been reported in the literature, the condition is considered extremely rare [3, 5]. Most cases have been reported in individuals born to consanguineous parents [3, 5]. However, the true population-level prevalence and carrier frequency of this disorder remain poorly defined.

The advent of large-scale genomic databases has improved the ability to estimate the prevalence of genetic diseases across diverse populations. To estimate the prevalence of AME, we used the gnomAD v4.1 database [6], a publicly available resource aggregating harmonized sequencing data from multiple large-scale projects, to calculate the global prevalence and carrier frequency of AME, with stratification by ethnic ancestry to identify potential disparities.

## Methods

### The gnomAD v4.1 database

We utilized the gnomAD v4.1 database (https://gnomad.broadinstitute.org/) [6], which includes aggregated genetic data from 730,947 exome and 76,215 whole-genome sequences derived from unrelated individuals. All data were mapped to the GRCh38 human reference genome, yielding a total of 807,162 samples. The dataset underwent rigorous quality control to remove low-quality sequences and flag potentially unreliable variants. Genetic ancestry was determined through principal component analysis of high-quality single nucleotide variants to determine genetic clusters aligned with geographic ancestry. Individual samples were subsequently assigned ancestry labels using a random forest classifier. The underlying methods and source code are publicly accessible and based on the framework established by the International Genome Sample Resource [6, 7].

### Identification of potentially disease-causing variants

We identified potentially disease-causing variants based on three criteria: (1) pathogenic (P) or likely pathogenic (LP) reported by the ClinVar database (accessed April 25th 2025) [8]; (2) variants reported in patients with AME through a systematic review of the published literature and (3) in silico predictions.

For systematic review, we searched the Pubmed and Embase databases from inception to April 2025 using search strategies incorporating terms related to “apparent mineralocorticoid excess”, “11β-Hydroxysteroid Dehydrogenase Type 2” or “*HSD11B2*”. The full search strategy is detailed in the Supplementary Material 1. We identified all articles that provided clear descriptions of *HSD11B2* variants associated with AME. Conference abstracts were also included only if they contained sufficient clinical and genetic data. Variants classified as benign or likely benign (B/LB) in ClinVar were excluded. Variants of uncertain significance (VUS) were included only if they were reported in the literature to be associated with AME. The review process was conducted independently by two investigators (N.C., C.K.) to ensure comprehensiveness and minimize bias.

For in silico prediction, we employed the following criteria: all nonsense or frameshift variants; indel variants with a CADD score >20 [9] and predicted to be “damaging” by the SIFT-indel tool (https://sift.bii.a-star.edu.sg/) [10]; splice-site variants predicted to be disruptive, with a SpliceAI score ≥ 0.8, CADD score >20 and |pangolin score| >0.2 [9, 11, 12]; and missense variants with a CADD score >20 [9], SIFT score < 0.05 [13], PolyPhen2 score >0.85 [14] and REVEL score >0.75 [15].

Variants reported in ClinVar or the literature were considered higher-confidence variants associated with AME, while variants identified solely through in silico prediction were considered lower-confidence.

### Estimation of prevalence and carrier frequency of apparent mineralocorticoid excess

We estimated the carrier frequency of AME by calculating the combined allele frequency of qualifying variants and dividing it by the total number of individuals in the dataset. Standard errors of proportion were calculated using the formula: $\:SE=\:\sqrt{\frac{p(1-p)}{N}}$, where ‘p’ is the sample proportion and ‘N’ is the sample size. To generate lower and upper estimates of carrier frequency, we applied two variant filtering strategies: a strict approach including only variants classified as pathogenic or likely pathogenic (P/LP) in ClinVar or reported in the literature; and a more liberal approach that additionally included variants predicted to be deleterious by in silico tools. Carrier frequency and corresponding standard errors were also calculated within each gnomAD ancestry group: African/African American (*n* = 37,545), Admixed American (*n* = 30,019), Ashkenazi Jewish (*n* = 14,804), East Asian (*n* = 22,448), Finnish (*n* = 32,026), Middle Eastern (*n* = 3,031), Non-Finnish European (*n* = 590,031), South Asian (*n* = 45,546), and Remaining/Other (*n* = 31,256). The Amish group (*n* = 456) was excluded from ancestry-stratified analyses due to insufficient sample size.

To estimate the expected disease prevalence of AME, we applied Hardy-Weinberg equilibrium, assuming random mating and no selection [16]. Prevalence was calculated using the formula: $\:\text{P}\text{r}\text{e}\text{v}\text{a}\text{l}\text{e}\text{n}\text{c}\text{e}={\left(\frac{p}{2}\right)}^{2}$ where *p* is the estimated carrier frequency [17]. The standard error of disease prevalence was approximated using the delta method, with the formula:

$\:{\text{S}\text{E}}_{\text{P}\text{r}\text{e}\text{v}\text{a}\text{l}\text{e}\text{n}\text{c}\text{e}}\approx\:\frac{p}{2}\times\:{\text{S}\text{E}}_{\text{C}\text{a}\text{r}\text{r}\text{i}\text{e}\text{r}\:\text{f}\text{r}\text{e}\text{q}\text{u}\text{e}\text{n}\text{c}\text{y}}$ where *p* is the estimated carrier frequency. Prevalence and standard errors were calculated both overall and stratified by ethnic ancestry group and variant filtering criteria. Computations and data visualizations were performed using RStudio (version 2024.9.0.375; RStudio, PBC, Boston, MA).

## Results

**Fig. 1:**
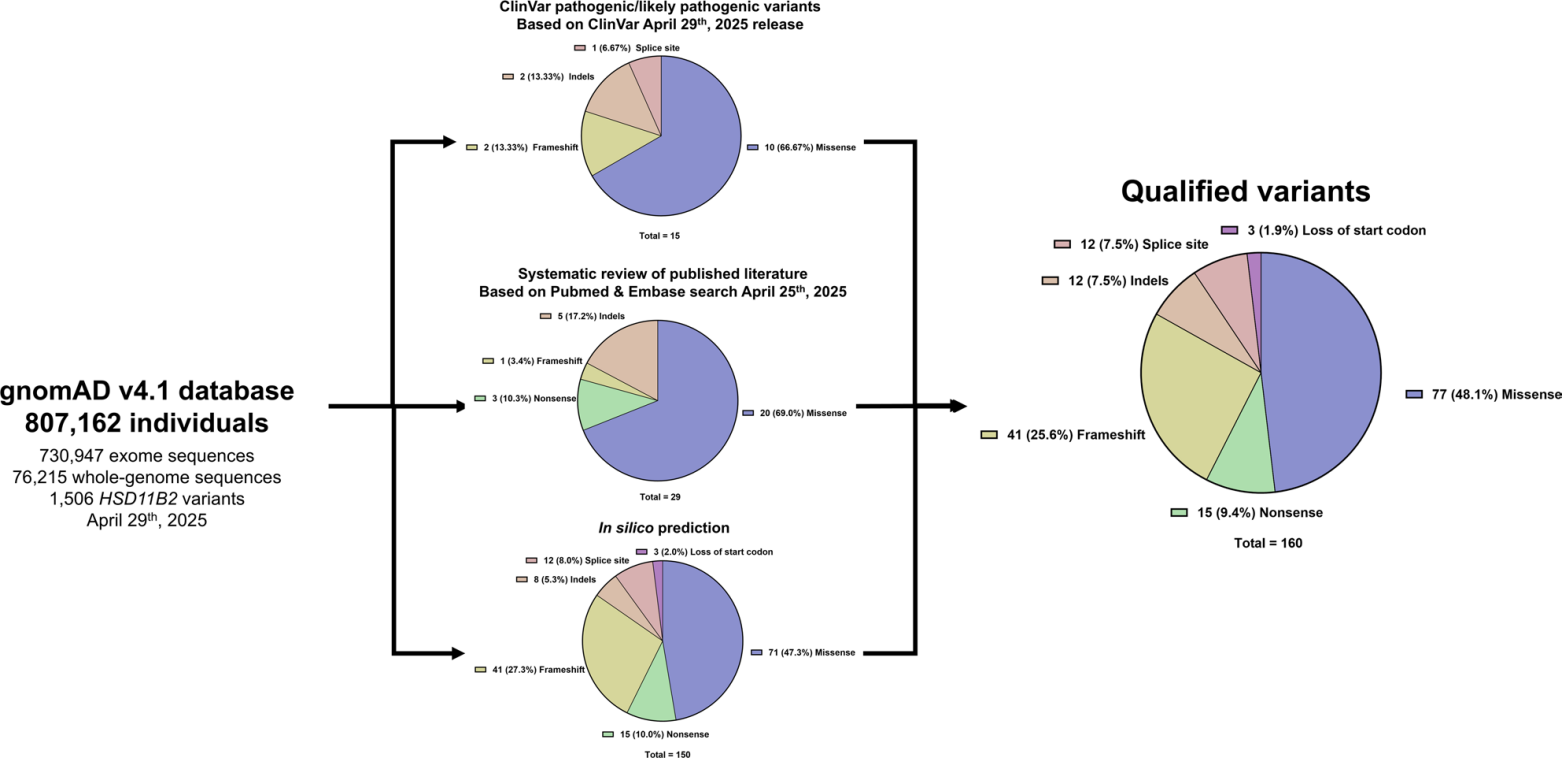
Summary of the variant identification process and proportions of qualifying variants by mutation type and filtering criteria

**Fig. 2:**
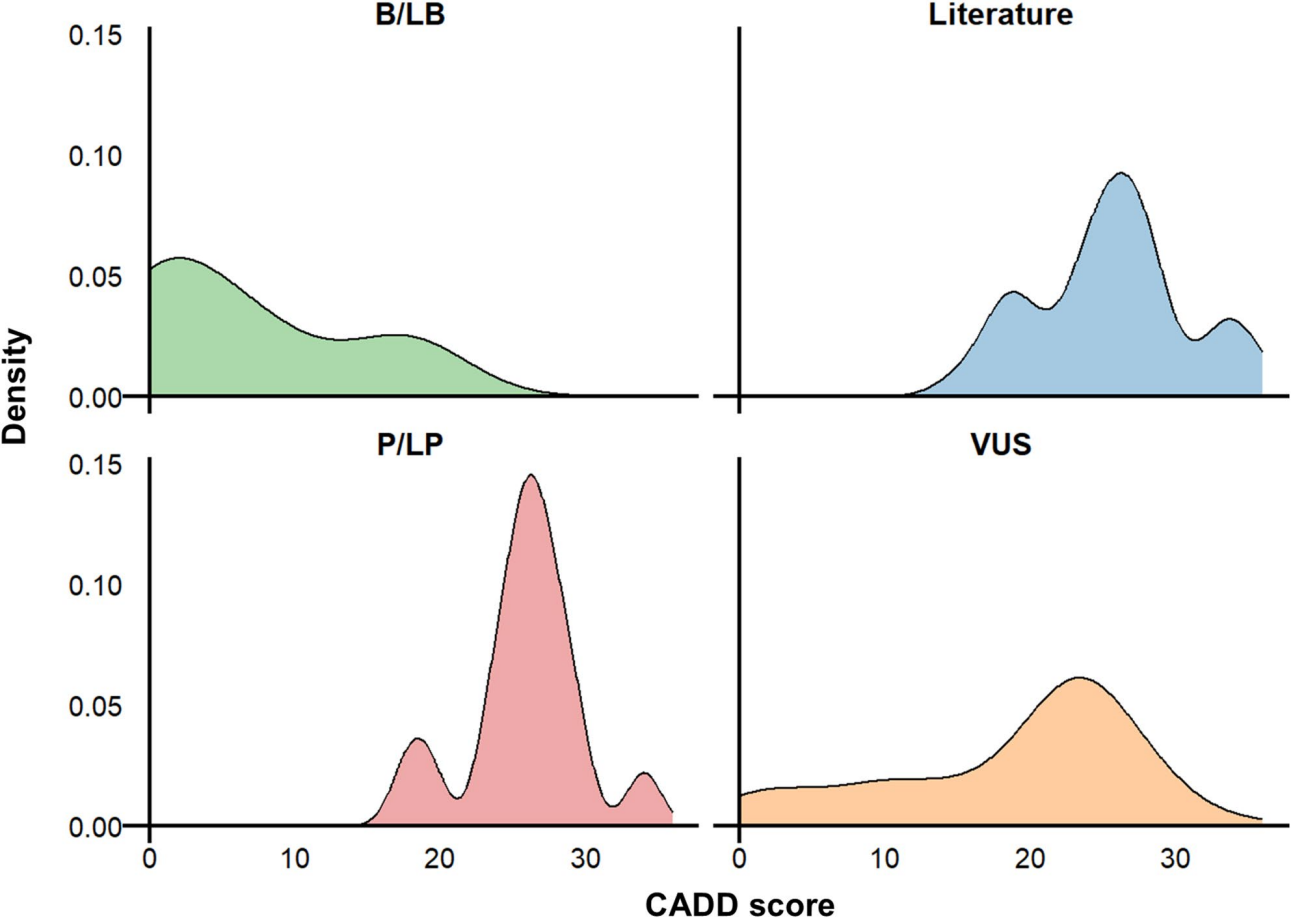
Distribution of CADD scores for *HSD11B2* variants in gnomAD, stratified by classification in ClinVar and by presence in the published literature

Allele frequency data were extracted from the gnomAD population, comprising 807,162 individuals of diverse ethnic ancestries. A total of 1,506 *HSD11B2* variants were analyzed, of which 160 were considered potentially associated with AME based on any of the three filtering criteria. Among these 160 variants, 15 were classified as pathogenic or likely pathogenic (P/LP) in ClinVar, 29 were reported in the literature, and 150 were predicted to be deleterious by in silico analysis. The literature review and variant identification process are summarized in Supplementary Fig. 1. The complete list of variants identified through systematic review, along with their references, is provided in Supplementary Table 1. Figure 1 summarizes the variant selection workflow and shows the distribution of qualified variants by mutation type. The distribution of CADD scores across variants in ClinVar (classified as B/LB, VUS or P/LP) or reported in the literature is shown in Fig. 2. Notably, variants reported in the literature and those classified as P/LP tend to have higher CADD scores. In contrast, VUS variants span a wide range of CADD scores, while most B/LB variants have lower CADD scores.

**Fig. 3:**
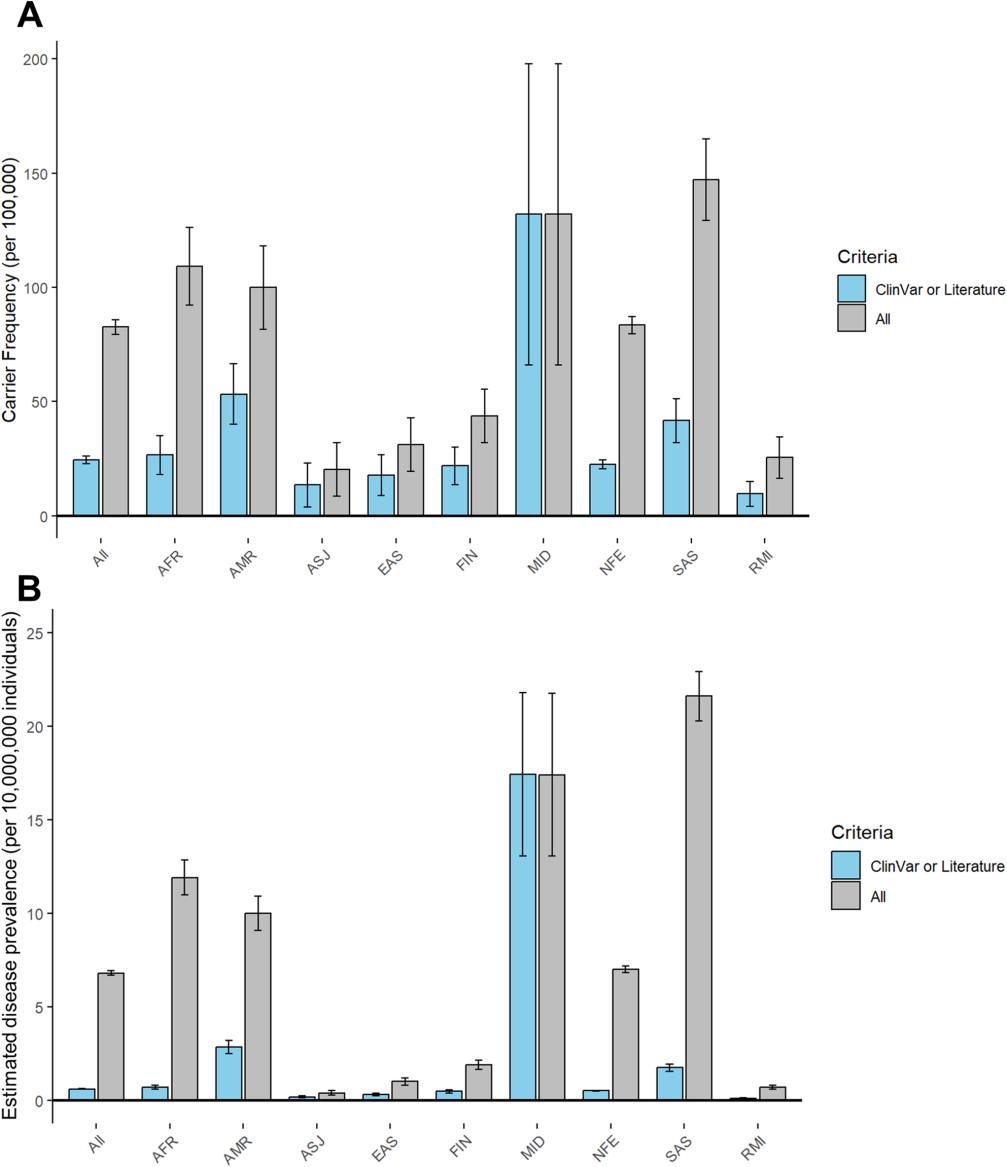
Estimated carrier frequency and Hardy-Weinberg-based disease prevalence of apparent mineralocorticoid excess, stratified by ethnic ancestry group. Data are presented as carrier frequency per 100,000 individuals and disease prevalence per 10,000,000 individuals, calculated using Hardy-Weinberg equilibrium. Error bars represent standard errors of the proportion. The left (blue) bars represent estimates based on the strict criteria, including only variants classified as pathogenic or likely pathogenic in ClinVar or reported in the literature. The right (gray) bars represent estimates based on the liberal criteria, which also include additional variants predicted to be deleterious by in silico tools. Abbreviations: AFR: African/African American; AMR: Admixed American; ASJ: Ashkenazi Jewish; EAS: East Asian; FIN: Finnish; MID: Middle Eastern; NFE: Non-Finnish European; SAS: South Asian; REM: Remaining

Estimated carrier frequency and disease prevalence, along with their standard errors, based on different variant selection criteria, are shown in Fig. 3 and Supplementary Table 2. Using the strict criteria (ClinVar or literature), the estimated carrier frequency in the overall gnomAD population was 24.5 per 100,000 individuals (Fig. 3A), corresponding to a disease prevalence of 0.6 per 10,000,000 individuals based on Hardy-Weinberg equilibrium (Fig. 3B). When applying the liberal criteria (ClinVar, literature or in silico analysis), the carrier frequency increased substantially to 82.6 per 100,000 individuals, with an estimated disease prevalence of 6.8 per 10,000,000 individuals, providing the upper bounds of these estimates. The highest estimated carrier frequency under the strict criteria was observed in the Middle Eastern group (132.0 per 100,000 individuals), whereas under the liberal criteria, the highest carrier frequency was found in the South Asian group (147.1 per 100,000 individuals) (Fig. 3A). Two individuals were identified as carrying homozygous variants classified as variants of uncertain significance (VUS) in ClinVar and predicted to be deleterious by in silico analysis. One individual of non-Finnish European ancestry carried the p.Ala39_Leu44dup variant, and another individual of South Asian ancestry carried the p.Tyr353His variant.

List of *HSD11B2* variants in the gnomAD v4.1 database reported in ClinVar or the literature sorted by allele count is shown in Table 1. Other variants identified by in silico analysis but not in ClinVar or literature are presented in Supplementary Table 3. The variant with highest allele count was p.Arg337_Tyr338delinsHis (allele count 20), followed by p.Arg359Trp (allele count 18), p.Leu376Pro (allele count 18), p.Asp223Asn (allele count 16) and p.Tyr232_Thr234del (allele count 16). Notably, certain *HSD11B2* variants were enriched in specific ethnic ancestry groups in gnomAD **(**Table 2**)**, such as p.Leu376Pro in the non-Finnish European population, p.Arg359Trp in South Asians, p.Asp223Asn in Admixed Americans, and p.Gly89Asp in the Middle Eastern group.

**Table 1:** List of *HSD11B2* variants in the gnomAD v4.1 database reported in Clinvar or the literature

Protein change	cDNA change	Variant type	ClinVar Germline Classification	ClinVar Variation ID	rsID	Allele count
*p.Arg337_Tyr338delinsHis	c.1010_1012del	Inframe deletion	P/LP	12,097	rs397509434	20
*p.Arg359Trp	c.1075 C > T	Missense	Not reported	-	rs373865007	18
*p.Leu376Pro	c.1127T > C	Missense	Not reported	-	rs762518964	18
*p.Asp223Asn	c.667G > A	Missense	VUS	12,101	rs121917833	16
*p.Tyr232_Thr234del	c.695_703del	Inframe deletion	LP	3,581,160	rs765985616	16
*p.Ala221Val	c.662 C > T	Missense	LP	3,581,156	rs1329450118	13
*p.Arg279Cys	c.835 C > T	Missense	P	12,098	rs28934594	13
*p.Tyr232Cys	c.695 A > G	Missense	Not reported	-	-	10
*p.Arg213Cys	c.637 C > T	Missense	P	12,094	rs28934591	8
*p.Ala237Val	c.710 C > T	Missense	VUS	800,834	rs1309642469	7
*p.Arg208His	c.623G > A	Missense	P/LP	12,096	rs28934592	7
*p.Ala328Val	c.983 C > T	Missense	LP	974,391	rs1453036708	6
*p.Arg337Cys	c.1009 C > T	Missense	P	12,095	rs121917781	5
*p.Tyr338His	c.1012T > C	Missense	P	31,131	rs387907117	5
*p.Arg208Cys	c.622 C > T	Missense	LP	12,093	rs121917780	4
*p.Gly89Asp	c.266G > A	Missense	Conflicting classifications	447,525	rs1555518481	4
*p.Arg374Ter	c.1120 C > T	Nonsense	Not reported	-	rs2040980585	3
*p.Tyr339Ter	c.1017 C > A	Nonsense	Not reported	-	rs2040979207	3
*p.Val255GlyfsTer102	c.763dup	Frameshift	Not reported	-	rs1453568208	3
*p.Glu115_Leu116del	c.343_348del	Inframe deletion	Conflicting classifications	12,100	rs794726669	2
*p.Met243Val	c.727 A > G	Missense	Not reported	-	-	2
*p.Phe367del	c.1099_1101del	Inframe deletion	Not reported	-	rs776630118	2
*p.Pro227Leu	c.680 C > T	Missense	P	12,099	rs121917782	2
*p.Ser26Ter	c.77 C > A	Nonsense	Not reported	-	-	2
p.Leu28ArgfsTer32	c.83_177del	Frameshift	P	2,033,856	rs2040931363	2
*p.Arg186Cys	c.556 C > T	Missense	LP	2,137,838	rs768507002	1
*p.Asp244Asn	c.730G > A	Missense	Not reported	-	-	1
*p.Cys90Arg	c.268T > C	Missense	Not reported	-	rs2040963761	1
*p.Leu363Pro	c.1088T > C	Missense	Not reported	-	-	1
*p.Tyr299del	c.895_897del	Inframe deletion	VUS	12,102	rs794726670	1
c.665-1G > A	-	Splice acceptor	LP	3,581,157	rs2040971502	1
p.Ala57GlyfsTer51	c.168_195del	Frameshift	P	2,876,313	rs2040932336	1

Abbreviations P, pathogenic; LP, likely pathogenic; VUS, variant of uncertain significance

* indicates variants reported in the literature. The remaining variants were reported only in ClinVar

**Table 2:** List of *HSD11B2* variants in the gnomAD v4.1 database enriched in specific ethnic ancestry groups

Ethnic ancestry	Protein change	cDNA change	Variant type	rsID	Allele count within ancestry group	Carrier frequency within ancestry group (per 100,000)
AFR	p.Leu284Arg	c.851T > G	Missense	rs1263624159	12	32.0
AFR	*p.Ala221Val	c.662 C > T	Missense	rs1329450118	3	8.0
AFR	p.Gly296Ser	c.886G > A	Missense	rs895642778	3	8.0
AFR	*p.Leu28ArgfsTer32	c.83_177del	Frameshift	rs2040931363	2	5.3
AFR	p.Asp317His	c.949G > C	Missense	rs147758873	2	5.3
AMR	*p.Asp223Asn	c.667G > A	Missense	rs121917833	13	43.3
AMR	p.Pro381LeufsTer15	c.1142del	Frameshift	rs1295704073	3	10.0
AMR	*p.Arg208His	c.623G > A	Missense	rs28934592	2	6.7
EAS	p.Phe107Ile	c.319T > A	Missense	rs749262459	2	8.9
MID	*p.Gly89Asp	c.266G > A	Missense	rs1555518481	4	132.0
NFE	*p.Leu376Pro	c.1127T > C	Missense	rs762518964	17	2.9
NFE	*p.Tyr232_Thr234del	c.695_703del	Inframe deletion	rs765985616	16	2.7
NFE	*p.Arg337_Tyr338delinsHis	c.1010_1012del	Inframe deletion	rs397509434	16	2.7
NFE	*p.Arg279Cys	c.835 C > T	Missense	rs28934594	11	1.9
NFE	*p.Tyr232Cys	c.695 A > G	Missense	-	10	1.7
NFE	*p.Arg213Cys	c.637 C > T	Missense	rs28934591	8	1.4
NFE	*p.Ala237Val	c.710 C > T	Missense	rs1309642469	5	0.8
NFE	*p.Arg337Cys	c.1009 C > T	Missense	rs121917781	5	0.8
NFE	*p.Ala328Val	c.983 C > T	Missense	rs1453036708	4	0.7
NFE	*p.Tyr338His	c.1012T > C	Missense	rs387907117	4	0.7
NFE	*p.Val255GlyfsTer102	c.763dup	Frameshift	rs1453568208	3	0.5
NFE	*p.Tyr339Ter	c.1017 C > A	Nonsense	rs2040979207	3	0.5
NFE	*p.Pro227Leu	c.680 C > T	Missense	rs121917782	2	0.3
NFE	*p.Met243Val	c.727 A > G	Missense	-	2	0.3
NFE	*p.Arg374Ter	c.1120 C > T	Nonsense	rs2040980585	2	0.3
SAS	p.Tyr353His	c.1057T > C	Missense	rs764653595	19	41.7
SAS	*p.Arg359Trp	c.1075 C > T	Missense	rs373865007	8	17.6
SAS	p.Phe367Ile	c.1099T > A	Missense	-	5	11.0
SAS	p.Leu114Ser	c.341T > C	Missense	rs770686551	4	8.8
SAS	-	c.803_804del	Splice acceptor	-	3	6.6
SAS	p.Gln387Ter	c.1159 C > T	Nonsense	-	2	4.4

Abbreviations: AFR: African/African American; AMR: Admixed American; EAS: East Asian; MID: Middle Eastern; NFE: Non-Finnish European; SAS: South Asian

* indicates variants reported in the literature or ClinVar. The rest of the variants were predicted to be potentially deleterious from in silico analysis. For non-Finnish European group, only variants reported in the literature or ClinVar were reported. Carrier frequency was calculated as allele count divided by number of individuals in the ethnic ancestry group

## Discussion

This study provides the first large-scale, population-based estimates of the carrier frequency and prevalence of AME across diverse ancestral groups. By analyzing genomic data from more than 800,000 individuals in the gnomAD v4.1 database, we identified significant variability in carrier frequencies depending on both ancestry and the criteria used to define potentially pathogenic variants. These findings offer new insights into the global distribution of AME-associated genetic risk and carry important clinical and research implications.

While AME remains clinically rare, our analysis demonstrates that the estimated carrier frequency in the general population may be higher than previously appreciated, particularly when broader variant inclusion criteria are applied. Using a strict definition based on ClinVar or literature-reported variants, the carrier frequency was estimated at 24.5 per 100,000 individuals. However, when including deleterious variants predicted by in silico tools, the estimated carrier frequency increased more than threefold to 82.6 per 100,000. These findings highlight how variant classification frameworks can substantially influence the perceived burden of this rare autosomal recessive disease.

Ethnic ancestry-specific analyses revealed notable disparities in carrier frequencies. The highest carrier frequency under the strict criteria was observed in the Middle Eastern population (132.0 per 100,000), suggesting that the higher number of reported cases in this group may be due not only to increased rates of consanguinity [18] but also to an intrinsically higher carrier frequency. The p.Gly89Asp variant, which appears enriched in this population, may represent a potential founder effect. However, given the relatively small sample size in the Middle Eastern group (*n* = 3,031), the confidence in this conclusion remains limited. Under the liberal criteria, the South Asian population exhibited the highest estimated carrier frequency (147.1 per 100,000), which may reflect true genetic enrichment or limitations in current variant curation and classification systems, particularly due to the limited availability of phenotypic data from South Asian populations.

Our study has a number of notable strengths. By utilizing the large and ethnically diverse gnomAD v4.1 database, we were able to estimate population-based carrier frequency and disease prevalence of AME in a relatively unbiased manner, addressing the limitations of earlier studies based primarily on case reports. Additionally, the incorporation of a systematic manual review of the literature improved confidence in variant pathogenicity classification under the strict criteria used to define qualifying variants. In contrast, the inclusion of deleterious variants predicted by in silico tools under the liberal criteria allowed us to estimate the upper bounds of disease prevalence, accounting for variants that may not yet be well characterized but could possibly contribute to disease risk. Functional studies are particularly warranted for variants classified as VUS, as highlighted in Supplementary Table 3 (e.g., p.Ala39_Leu44dup, p.Tyr353His, p.Leu318Pro). Such data will be clinically important, as they may help refine pathogenicity classification and improve diagnostic accuracy of genetic testing for AME.

Several limitations should also be noted. First, the gnomAD dataset may exclude individuals with severe pediatric disease and related individuals [19], potentially leading to underestimation of homozygous cases and overall disease prevalence. Second, our disease prevalence estimates rely on Hardy-Weinberg equilibrium assumptions [16], which may not hold in populations with high consanguinity. Additionally, although prevalence was estimated within each ancestry group, these estimates may be less accurate in admixed populations where parents have different ancestries, potentially violating equilibrium assumptions. Third, the absence of phenotypic data in gnomAD precludes the assessment of genotype-phenotype relationships and disease penetrance which can impact clinical interpretability. In this study, we assumed that AME is fully penetrant in individuals carrying biallelic inactivating *HSD11B2* variants. In reality, incomplete penetrance, variable expressivity, and environmental modifiers may influence the proportion of genetically predisposed individuals who develop clinical manifestations. It is also unclear whether individuals heterozygous for pathogenic variants exhibit milder phenotypes such as hypertension. However, a phenome-wide association study of the *HSD11B2* gene using data from the AstraZeneca PheWAS Portal (https://azphewas.com/) did not demonstrate significant associations with hypertension or related traits [20]. Finally, while gnomAD provides a powerful and broad dataset, certain populations remain underrepresented as these sample sizes in some ancestry groups are smaller than others, such as the Middle Eastern and Ashkenazi Jewish populations. This potentially skews ancestry-specific frequencies and may limit the precision of prevalence estimates in those groups.

## Conclusion

This study provides the first large-scale, global estimates of AME carrier frequency and disease prevalence, revealing substantial variability influenced by both ethnic ancestry and variant classification criteria. The marked discrepancy between carrier frequency and disease prevalence estimates derived from strict criteria based on ClinVar or literature data, and liberal criteria that include in silico-predicted variants, highlights gaps in current variant curation that warrant further functional and clinical investigation. Further research integrating genomic and phenotypic data, along with better representation of diverse populations in genetic databases, will be critical to improving our understanding of the genetic epidemiology of AME.

## Supplementary Information

Below is the link to the electronic supplementary material.

Supplementary Material 1

## Data Availability

The data used in this study are publicly available from the Genome Aggregation Database (gnomAD) version 4.1 (https://gnomad.broadinstitute.org/). All variant frequency data, including ancestry-stratified information, were obtained from this resource. The specific variant filtering criteria, classification methods, and code used for analysis are available from the corresponding author upon reasonable request.
