# Supplementary material for: Population-based estimates of the global prevalence and carrier frequency of apparent mineralocorticoid excess caused by 11β-hydroxysteroid dehydrogenase type 2 deficiency
Source: Orphanet J Rare Dis. 2025 Nov 28;21:1. doi: 10.1186/s13023-025-04160-x (PMC12764048; doi:10.1186/s13023-025-04160-x)
Supplement: Supplementary file 1 — Supplementary Material 1 [file 13023_2025_4160_MOESM1_ESM.docx]

**Supplementary Material 1** – Search strategies to identify HSD11B2 variants reported in the literature

**Pubmed (searched April 25, 2025)**

"apparent mineralocorticoid excess"[Title/Abstract] OR

"11beta-hydroxysteroid dehydrogenase type 2"[Title/Abstract] OR

"HSD11B2"[Title/Abstract] OR

"11beta-HSD2"[Title/Abstract]

**Embase (searched April 25, 2025)**

1. 'apparent mineralocorticoid excess syndrome'/exp OR 'apparent mineralocorticoid excess syndrome'
2. apparent AND ('mineralocorticoid'/exp OR mineralocorticoid) AND excess
3. hsd11b2
4. '11beta hydroxysteroid dehydrogenase 2'/exp OR '11beta hydroxysteroid dehydrogenase 2'
5. '11beta hsd2 gene'/exp OR '11beta hsd2 gene'
6. '11beta hsd2'/exp OR '11beta hsd2'
7. #1 OR #2 OR #3 OR #4 OR #5 OR #6

**Supplementary Figure 1 –** Summary of the literature review and variant identification process


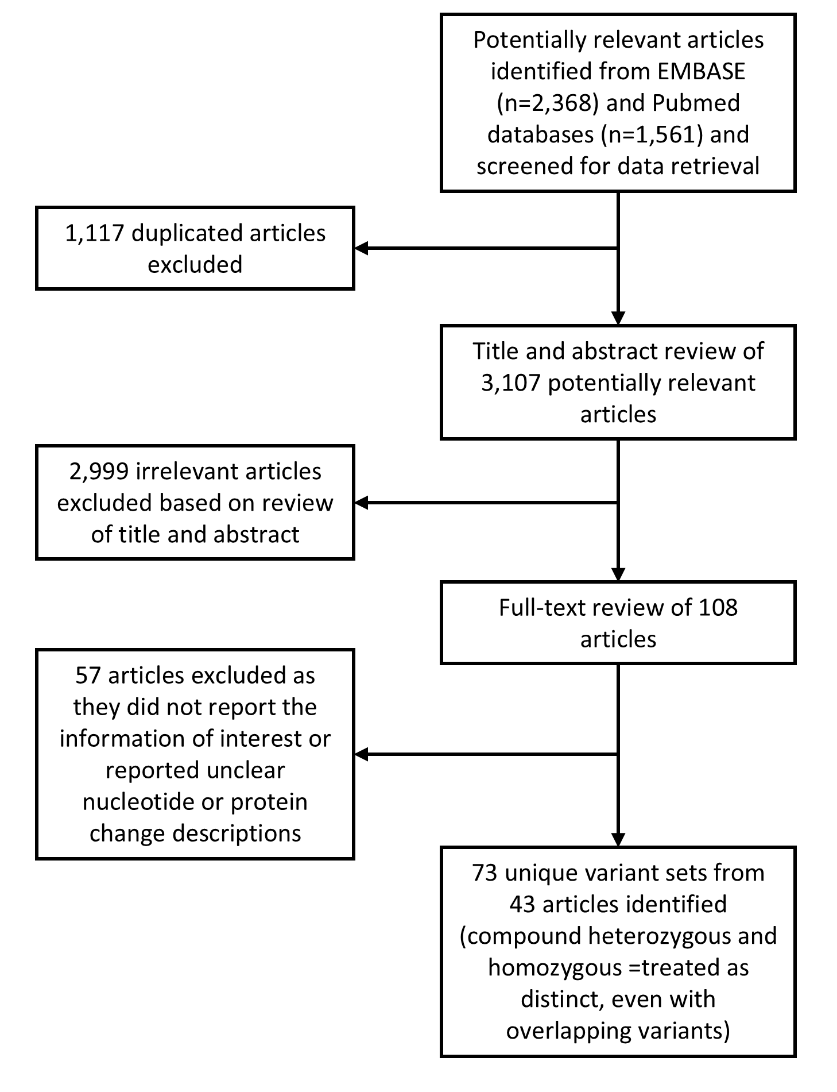


**Supplementary Table 1. List of variants reported in the literature**

| **Variant** | **ClinVar pathogenicity** | **Variant type** | **Country/ethnicity of patient** |
| --- | --- | --- | --- |
| c.IVS3+14C>T, p.Asp223Asn | -/VUS | Intronic, Missense | Chilean (1) |
| c.IVS3+14C>T, p.Asp223Asn/p.Arg337_Tyr338delinsHis | -/- | Intronic/Missense* | Not Specified (2) |
| p.Ala221Gly | - | Missense | Italian (3, 4) |
| p.Ala221Val | LP | Missense | Omani (4-6) |
| p.Ala221Val/p.Tyr339Ter | LP, - | Missense/Nonsense* | Chinese (7) |
| p.Ala237Val | VUS | Missense | Not specified (3, 7) |
| p.Ala237Val/p.Ala328Val | -/LP | Missense/Missense* | Not Specified (8) |
| p.Ala328Val | LP | Missense | Portugese (9), Brazilian (10) |
| p.Ala59Valfs*28 | - | Frameshift | Spain (11) |
| p.Arg186Cys | LP | Missense | African American(4), Mennonite (12), Brazilian (13) |
| p.Arg208Cys | LP | Missense | Saudi Arabian (14), Omani (4, 15), Mennonite (16), Middle Eastern (12), Native American (12, 17) |
| p.Arg208His | P or LP | Missense | Turkey (18), Not specified (19) |
| p.Arg208His/p.Arg337_Tyr338delinsHis | P or LP | Missense/Indel* | Japanese (20) |
| p.Arg213Cys | P | Missense | South American:(17), Chilean(21, 22), Algeria (9), French (9) Argentina: (23) |
| p.Arg279Cys | P | Missense | Italian (24) |
| p.Arg328Val | LP | Missense | Portuguese (25), Brazilian (12) |
| p.Arg337_Tyr338delinsHis | P or LP | Indel | Persian (4), Indian (12, 15, 26), Iranian(15), Menonite (16) Zoroastrian (12) |
| p.Arg337Cys | P | Missense | Iranian (4, 15, 27), Indian (17, 28), Mennonite (16), Not specified (29, 30) |
| p.Arg359Trp/IVS3+1G>A | - | Missense/splice-site* | Italian (31) |
| p.Arg374Ter | - | Nonsense | Pakistani (12), Asian (32), Caucasian (12), Not specified (30, 33) |
| p.Arg74Gly | - | Missense | Not specified (3) |
| p.Arg74Gly/p.Pro75delC | - | Missense/Frameshift* | Omani (6) |
| p.Arg74Glyfs*43 | - | Frameshift | Omani (4) |
| p.Asn286fs | - | Frameshift | Turkish (15) |
| p.Asp144Val | - | Missense | Not Specified (3, 4) |
| p.Asp144Val/p.Phe367del | -/- | Missense/In-frame deletion* | French (25) |
| p.Asp176Asn | - | Missense | Saudi Arabia (34) |
| p.Asp223Asn | VUS | Missense | Not Specified (3, 4) |
| p.Asp244Asn/p.Leu250Arg | -/- | Missense/Missense* | Italian-Moroccan (12), Mennonite (16), Italian (4) |
| p.Asp244Asn | - | Missense | Not Specified (3, 4) |
| p.Cys271Gly | - | Missense | Not Specified (3) |
| p.Cys90Arg | - | Missense | Arabic (35) |
| p.Gln342Profs*62 | - | Frameshift | Not Specified (3, 4) |
| p.Glu115_Leu116del | P or VUS | In-frame deletion | Omani (4) |
| p.Glu301Argfs*56 | - | Frameshift | Pakistani (36) |
| p.Glu342fs | - | Frameshift | Morocco (25) |
| p.Glu356Valfs*40 | - | Frameshift | American Indian (4, 12) |
| p.Gly290Ala | - | Missense | Arabic (35) |
| p.Gly305Glufs* | - | Frameshift | Not Specified (4) |
| p.Gly341Ser/p.His304Arg | VUS/- | Missense/Missense* | Russian (37) |
| p.Gly89Asp | LP | Missense | Qatari (38), Saudi Arabian (39) |
| p.Leu114_Glu115del | - | In-frame deletion | Omani (6, 40) |
| p.Leu179Arg | - | Missense | Not Specified (3, 4) |
| p.Leu250_Leu251delinsProSer | - | In-frame deletion | Native American (4, 15, 17), Mennonite (16) |
| p.Leu250Arg | - | Missense | Not Specified (3, 4) |
| p.Leu250Arg/p.Asp244Asn | -/- | Missense/Missense* | Italian-Moroccan (12) |
| p.Leu287Cysfs*36 | - | Frameshift | Australian (Turkish) (4) |
| p.Leu363Pro | - | Missense | Chinese (41) |
| p.Leu376Pro | - | Missense | Not Specified (3, 4) |
| p.Leu395Ter | - | Nonsense | Not Specified (3) |
| p.Met1Val;del85_179 | - | Frameshift | Italian (3) |
| p.Met243Val | - | Missense | Russian (37) |
| p.Phe185Ser | - | Missense | Turkish (42), French Guyana (25) |
| p.Phe246fs* | - | Splice site | Not Specified (4) |
| p.Phe367del | - | In-frame deletion | Not Specified (4) |
| p.Phe367fs* | - | Frameshift | Not specified (3) |
| p.Pro227Leu | P | Missense | Mennonite (4, 12, 16) |
| p.Ser180Phe | - | Missense | Not Specified (3, 4) |
| p.Ser26Ter | - | Nonsense | Not Specified (4) |
| p.Thr267Ala | - | Missense | Omani (4, 5) |
| p.Tyr226Asn | - | Missense | Not Specified (3, 4) |
| p.Tyr226Asp/c.771C>G | - | Missense/Splice site* | Not Specified (31) |
| p.Tyr232_Thr234del | LP | In-frame deletion | Not Specified (3) |
| p.Tyr232Cys | - | Missense | Not Specified (4) |
| p.Tyr232Cys/p.Leu376Pro | -/- | Missense/Missense* | Not Specified (31) |
| p.Tyr295Ter | - | Nonsense | Brazilian (43) |
| p.Tyr299del | VUS | In-frame deletion | Pakistani (4) |
| p.Tyr338His | P | Missense | Not Specified (3, 4) |
| p.Val217Ala | - | Missense | Chinese (7) |
| p.Val255Glyfs*102/p.Leu69Alafs*15 | - | Frameshift/Frameshift* | Chinese (7) |
| p.Val257Serfs*3 | - | Frameshift | Not Specified (4) |
| p.Val321_Val322insAlaProVal | VUS | In-frame insertion | Omani (4) |
| p.Val322_Val323insProGlnLeu | - | In-frame insertion | Omani (6) |

**Abbreviations:** P, pathogenic; LP, likely pathogenic; VUS, variant of uncertain significance. *Denotes compound heterozygous mutations. All other mutations reported are homozygous or not specified.

References

1. Carvajal CA, Gonzalez AA, Romero DG, González A, Mosso LM, Lagos ET, et al. Two homozygous mutations in the 11β-hydroxysteroid dehydrogenase type 2 gene in a case of apparent mineralocorticoid excess. Journal of Clinical Endocrinology and Metabolism. 2003;88(6):2501-7.

2. Alonso G, Marino R, Perez Garrido N, Ramírez P, Blazquez J, Ghezzi L, et al. Apparent mineralocorticoid excess: Case report and molecular diagnosis. Hormone Research in Paediatrics. 2012;78:14.

3. Pizzolo F, Friso S, Morandini F, Antoniazzi F, Zaltron C, Udali S, et al. Apparent mineralocorticoid excess by a novel mutation and epigenetic modulation by HSD11B2 promoter methylation. 2015. p. E1234-E41.

4. Yau M, Haider S, Khattab A, Ling C, Mathew M, Zaidi S, et al. Clinical, genetic, and structural basis of apparent mineralocorticoid excess due to 11β-hydroxysteroid dehydrogenase type 2 deficiency. Proceedings of the National Academy of Sciences of the United States of America. 2017;114(52):E11248-E56.

5. Yau M, Azkawi HSA, Haider S, Khattab A, Badi MA, Abdullah W, et al. A novel mutation in HSD11B2 causes apparent mineralocorticoid excess in an Omani kindred. Annals of the New York Academy of Sciences. 2016;1376(1):65-71.

6. Quinkler M, Bappal B, Draper N, Atterbury AJ, Lavery GG, Walker EA, et al. Molecular basis for the apparent mineralocorticoid excess syndrome in the Oman population. Mol Cell Endocrinol. 2004;217(1-2):143-9.

7. Ding Y, Cheng M, Cao B, Liu M, Hu X, Wu D. Case report: Clinical characteristics and Genetical analysis of HSD11B2 in three Chinese children with apparent mineralocorticoid excess: a case series. Front Endocrinol (Lausanne). 2024;15:1491825.

8. Moudgil A, Rodich G, Jordan SC, Kamil ES. Nephrocalcinosis and renal cysts associated with apparent mineralocorticoid excess syndrome. Pediatr Nephrol. 2000;15(1-2):60-2.

9. Morineau G, Marc JM, Boudi A, Galons H, Gourmelen M, Corvol P, et al. Genetic, biochemical, and clinical studies of patients with A328V or R213C mutations in 11βHSD2 causing apparent mineralocorticoid excess. Hypertension. 1999;34(3):435-41.

10. Li A, Li KXZ, Marui S, Krozowski ZS, Batista MC, Whorwood CB, et al. Apparent mineralocorticoid excess in a Brazilian kindred: Hypertension in the heterozygote state. Journal of Hypertension. 1997;15(12 I):1397-402.

11. Hernández Tejedor C, Romero Salas Y, Miramar Gallart MD, Bríngola Moñux AJ, Sánchez Malo MJ, Peña Segura JL. A case report of apparent mineralocorticoid excess, with nephrological and neurological symptoms since birth, and with a new probably pathogenic variant in HSD11B2 gene. Nefrologia (Engl Ed). 2024;44(6):904-6.

12. Wilson RC, Dave-Sharma S, Wei JQ, Obeyesekere VR, Li K, Ferrari P, et al. A genetic defect resulting in mild low-renin hypertension. Proceedings of the National Academy of Sciences of the United States of America. 1998;95(17):10200-5.

13. Coeli FB, Ferraz LFC, De Lemos-Marini SHV, Rigatto SZP, Belangero VMS, Maricilda PDM. Apparent mineralocorticoid excess syndrome in a Brazilian boy caused by the homozygous missense mutation p.R186C in the HSD11B2 gene. Arquivos Brasileiros de Endocrinologia e Metabologia. 2008;52(8):1277-81.

14. Al-Harbi T, Al-Shaikh A. Apparent mineralocorticoid excess syndrome: report of one family with three affected children. J Pediatr Endocrinol Metab. 2012;25(11-12):1083-8.

15. Dave-Sharma S, Wilson RC, Harbison MD, Newfield R, Azar MR, Krozowski ZS, et al. Examination of genotype and phenotype relationships in 14 patients with apparent mineralocorticoid excess. Journal of Clinical Endocrinology and Metabolism. 1998;83(7):2244-54.

16. Ugrasbul F, Wiens T, Rubinstein P, New MI, Wilson RC. Prevalence of mild apparent mineralocorticoid excess in Mennonites. J Clin Endocrinol Metab. 1999;84(12):4735-8.

17. Mune T, Rogerson FM, Nikkila H, Agarwal AK, White PC. Human hypertension caused by mutations in the kidney isozyme of 11β- hydroxysteroid dehydrogenase. Nature Genetics. 1995;10(4):394-9.

18. Gulhan B, Ünsal Y, Baltu D, Çelik Ertaş NB, Özdemir G, Utine E, et al. Apparent mineralocorticoid excess: A diagnosis beyond classical causes of severe hypertension in a child. Blood Press Monit. 2022;27(3):208-11.

19. Thakkar K, Yau M, Romero C. Hypertension of Cushing's Disease Masked by Apparent Mineralocorticoid Excess. Hormone Research in Paediatrics. 2022;95:161-2.

20. Kitanaka S, Katsumata N, Tanae A, Hibi I, Takeyama KI, Fuse H, et al. A new compound heterozygous mutation in the 11β-hydroxysteroid dehydrogenase type 2 gene in a case of apparent mineralocorticoid excess. Journal of Clinical Endocrinology and Metabolism. 1997;82(12):4054-8.

21. Pinochet C, Carvajal C, Godoy A, Lacourt P, Fardella C, Godoy C. Severe hypertension in a girl: Cushing syndrome or apparent mineralocorticoid excess syndrome? Utility of molecular study. Hormone Research in Paediatrics. 2015;84:16-7.

22. Rodríguez JA. [Syndrome of apparent mineralocorticoid excess caused by a deficiency of 11 beta-hydroxysteroid dehydrogenase: clinical and genetic study in a Chilean family followed for 19 years]. Rev Med Chil. 2000;128(1):17-26.

23. Rogoff D, Smolenicka Z, Bergadá I, Vallejo G, Barontini M, Heinrich JJ, et al. The codon 213 of the 11β-hydroxysteroid dehydrogenase type 2 gene is a hot spot for mutations in apparent mineralocorticoid excess. Journal of Clinical Endocrinology and Metabolism. 1998;83(12):4391-3.

24. Palermo M, Delitala G, Sorba G, Cossu M, Satta R, Tedde R, et al. Does kidney transplantation normalise cortisol metabolism in apparent mineralocorticoid excess syndrome? Journal of Endocrinological Investigation. 2000;23(7):457-62.

25. Morineau G, Sulmont V, Salomon R, Fiquet-Kempf B, Jeunemaître X, Nicod J, et al. Apparent mineralocorticoid excess: report of six new cases and extensive personal experience. J Am Soc Nephrol. 2006;17(11):3176-84.

26. Khattab AM, Shackleton CHL, Hughes BA, Bodalia JB, New MI. Remission of hypertension and electrolyte abnormalities following renal transplantation in a patient with apparent mineralocorticoid excess well documented throughout childhood. Journal of Pediatric Endocrinology and Metabolism. 2014;27(1-2):17-21.

27. Razzaghy-Azar M, Yau M, Khattab A, New MI. Apparent mineralocorticoid excess and the long term treatment of genetic hypertension. J Steroid Biochem Mol Biol. 2017;165(Pt A):145-50.

28. Narayanan R, Karuthedath Vellarikkal S, Jayarajan R, Verma A, Dixit V, Scaria V, et al. Case Report: Application of whole exome sequencing for accurate diagnosis of rare syndromes of mineralocorticoid excess. F1000Research. 2017;5.

29. Obeyesekere VR, Ferrari P, Andrews RK, Wilson RC, New MI, Funder JW, et al. The R337C mutation generates a high km 11β-hydroxysteroid dehydrogenase type II enzyme in a family with apparent mineralocorticoid excess. Journal of Clinical Endocrinology and Metabolism. 1995;80(11):3381-3.

30. Krozowski ZS, Stewart PM, Obeyesekere VR, Li K, Ferrari P. Mutations in the 11β-hydroxysteroid dehydrogenase type II enzyme associated with hypertension and possibly stillbirth. Clinical and Experimental Hypertension. 1997;19(5-6):519-29.

31. Lavery GG, Ronconi V, Draper N, Rabbitt EH, Lyons V, Chapman KE, et al. Late-onset apparent mineralocorticoid excess caused by novel compound heterozygous mutations in the HSD11B2 gene. Hypertension. 2003;42(2):123-9.

32. Stewart PM, Krozowski ZS, Gupta A, Milford DV, Howie AJ, Sheppard MC, et al. Hypertension in the syndrome of apparent mineralocorticoid excess due to mutation of the 11β-hydroxysteroid dehydrogenase type 2 gene. Lancet. 1996;347(8994):88-91.

33. Whorwood CB, Stewart PM. Human hypertension caused by mutations in the 11β-hydroxysteroid dehydrogenase gene: a molecular analysis of apparent mineralocorticoid excess. Journal of Hypertension, Supplement. 1996;14(5):S19-S24.

34. Alzahrani AS, Aljuhani N, Qasem E, Almohanna M, Hamoudh E, AlOmair A, et al. Apparent Mineralocorticoid Excess Caused by a Novel Mutation in 11-β Hydroxysteroid Dehydrogenase Type 2 Enzyme: Its Genetics and Response to Therapy. Endocr Pract. 2014;20(9):e151-6.

35. Lebel A, Ben Shalom E, Mokatern R, Halevy R, Zehavi Y, Magen D. Apparent mineralocorticoid excess in Israel: a case series and literature review. Eur J Endocrinol. 2024;190(5):347-53.

36. Bertulli C, Hureaux M, De Mutiis C, Pasini A, Bockenhauer D, Vargas-Poussou R, et al. A Rare Cause of Chronic Hypokalemia with Metabolic Alkalosis: Case Report and Differential Diagnosis. Children (Basel). 2020;7(11).

37. Makretskaya N, Kostrova I, Tiulpakov A. Two cases of apparent mineralocorticoid excess due to novel mutations in HSD11B2 gene. Hormone Research in Paediatrics. 2018;90:145.

38. Zahraldin K, Janahi IA, Ben-Omran T, Alsulaiman R, Hamad B, Imam A. Two Qatari siblings with cystic fibrosis and apparent mineralocorticoid excess. Ann Thorac Med. 2015;10(1):69-72.

39. Sawaf H, Wang X, Nakhoul G, Taliercio J. A Case of Apparent Mineralocorticoid Excess due to a Homozygous Rare Variant in Hydroxysteroid 11-Beta Dehydrogenase 2 Gene. American Journal of Kidney Diseases. 2022;79(4):S101.

40. Odermatt A, Dick B, Arnold P, Zaehner T, Plueschke V, Deregibus MN, et al. A mutation in the cofactor-binding domain of 11β-hydroxysteroid dehydrogenase type 2 associated with mineralocorticoid hypertension. Journal of Clinical Endocrinology and Metabolism. 2001;86(3):1247-52.

41. Wang Y, Ma L, Shu X, Li J, Hu J, Law KP, et al. Apparent mineralocorticoid excess caused by a novel mutation in 11β-hydroxysteroid dehydrogenase type 2 gene. J Hypertens. 2017;35(3):647-50.

42. LeventoǦlu E, Döǧer E, Büyükkaragöz B, Nalçaci S, Öner G, Alpman BN, et al. LATE-ONSET HYPERTENSION IN A CHILD WITH SYNDROME OF AME AND COFFIN-SIRIS. Pediatric Nephrology. 2022;37(11):2927.

43. Bachega TA, Rodrigues AS, Biscolla RPM, Mion D, Bronstein MD. A case of apparent mineralocorticoid excess responsible to dexamethasone caused by a novel HSD11B2 mutation. Endocrine Reviews. 2017;38(3).

Supplementary table 2. Estimated carrier frequency per 100,000 population (95% CI) and standard error (SE) stratified by ancestry group and variant classification criteria

| **Ethnicity** | **ClinVar P/LP (per 100k, 95% CI)** | **SE** | **Literature  (per 100k, 95% CI)** | **SE** | ***In silico*  (per 100k, 95% CI)** | **SE** | **All  (per 100k, 95% CI)** | **SE** |
| --- | --- | --- | --- | --- | --- | --- | --- | --- |
| All | 12.9 (10.4–15.4) | 1.26 | 24.0 (20.7–27.4) | 1.73 | 72.1 (66.2–78.0) | 2.99 | 82.6 (76.4–88.9) | 3.20 |
| AFR | 18.6 (4.8–32.5) | 7.05 | 21.3 (6.5–36.1) | 7.53 | 103.9 (71.3–136.5) | 16.62 | 109.2 (75.8–142.6) | 17.05 |
| AMR | 6.7 (-2.6–15.9) | 4.71 | 53.3 (27.2–79.4) | 13.32 | 46.6 (22.2–71.1) | 12.46 | 99.9 (64.2–135.7) | 18.24 |
| ASJ | 6.8 (-6.5–20.0) | 6.75 | 13.5 (-5.2–32.2) | 9.55 | 20.3 (-2.7–43.2) | 11.70 | 20.3 (-2.7–43.2) | 11.70 |
| EAS | 8.9 (-3.4–21.3) | 6.30 | 17.8 (0.4–35.3) | 8.91 | 26.7 (5.3–48.1) | 10.91 | 31.2 (8.1–54.3) | 11.78 |
| FIN | 18.7 (3.7–33.7) | 7.65 | 21.9 (5.7–38.0) | 8.26 | 37.5 (16.3–58.7) | 10.81 | 43.7 (20.8–66.6) | 11.68 |
| MID | 0.0 (0.0–0.0) | 0.00 | 132.0 (2.7–261.2) | 65.94 | 132.0 (2.7–261.2) | 65.94 | 132.0 (2.7–261.2) | 65.94 |
| NFE | 13.1 (10.1–16.0) | 1.49 | 22.2 (18.4–26.0) | 1.94 | 74.9 (67.9–81.9) | 3.56 | 83.6 (76.2–90.9) | 3.76 |
| SAS | 15.4 (4.0–26.8) | 5.81 | 41.7 (23.0–60.5) | 9.57 | 118.6 (87.0–150.2) | 16.12 | 147.1 (111.9–182.3) | 17.96 |
| RMI | 6.4 (-2.5–15.3) | 4.52 | 9.6 (-1.3–20.5) | 5.54 | 25.6 (7.9–43.3) | 9.05 | 25.6 (7.9–43.3) | 9.05 |

Abbreviations: 95% CI: 95% confidence interval of proportion (per 100,000); SE: standard error of proportion; AFR: African/African American; AMR: Admixed American; ASJ: Ashkenazi Jewish; EAS: East Asian; FIN: Finnish; MID: Middle Eastern; NFE: Non-Finnish European; SAS: South Asian; RMI: Remaining; P: Pathogenic; LP: Likely Pathogenic.

**Supplementary Table 3.** Variants predicted to be potentially deleterious by in silico tools but not reported as pathogenic or likely pathogenic in ClinVar or present the literature

| **Protein change** | **cDNA change** | **Variant type** | **Allele count** |
| --- | --- | --- | --- |
| *p.Ala39_Leu44dup | c.108_125dup | Inframe insertion | 87 |
| p.Val109Leu | c.325G>C | Missense | 57 |
| *p.Tyr353His | c.1057T>C | Missense | 22 |
| p.Ala121Thr | c.361G>A | Missense | 19 |
| p.Asn171MetfsTer3 | c.512del | Frameshift | 13 |
|  | c.266-1G>C | Splice acceptor | 13 |
| p.Leu284Arg | c.851T>G | Missense | 12 |
| *p.Leu318Pro | c.953T>C | Missense | 12 |
|  | c.801_802+2dup | Frameshift | 11 |
| p.Val215Ala | c.644T>C | Missense | 10 |
| *p.Gly341Ser | c.1021G>A | Missense | 10 |
| p.Met189Ile | c.567G>A | Missense | 8 |
| p.Met1? | c.1A>G | Loss of start codon | 7 |
| p.Leu114Ser | c.341T>C | Missense | 6 |
| p.Gly296Ser | c.886G>A | Missense | 6 |
| p.Arg213His | c.638G>A | Missense | 5 |
| p.Ser235Cys | c.704C>G | Missense | 5 |
| p.Asp298His | c.892G>C | Missense | 5 |
| p.Phe367Ile | c.1099T>A | Missense | 5 |
| p.Gln387Ter | c.1159C>T | Nonsense | 5 |
| p.Val165Leu | c.493G>C | Missense | 4 |
| p.Thr200Ile | c.599C>T | Missense | 4 |
| p.Trp4Ter | c.11G>A | Nonsense | 4 |
| p.Arg83ProfsTer36 | c.241_245dup | Frameshift | 3 |
| p.Leu206CysfsTer10 | c.612del | Frameshift | 3 |
| p.Pro381LeufsTer15 | c.1142del | Frameshift | 3 |
| p.Gly106Ala | c.317G>C | Missense | 3 |
| p.Phe107Ile | c.319T>A | Missense | 3 |
| p.Ala168Thr | c.502G>A | Missense | 3 |
| p.Asp317His | c.949G>C | Missense | 3 |
|  | c.803_804del | Splice acceptor | 3 |
| p.Gly31AlafsTer86 | c.92del | Frameshift | 2 |
| p.Leu51ArgfsTer62 | c.152_164del | Frameshift | 2 |
| p.Arg71IlefsTer47 | c.210_211insAT | Frameshift | 2 |
| p.Gln76HisfsTer40 | c.228_231del | Frameshift | 2 |
| p.Gly212AlafsTer4 | c.635del | Frameshift | 2 |
| p.Ser247GlnfsTer3 | c.737dup | Frameshift | 2 |
| p.Gly398AlafsTer71 | c.1191del | Frameshift | 2 |
| p.Ala404GlyfsTer75 | c.1210dup | Frameshift | 2 |
| p.Pro79_Ala81del | c.235_243del | Inframe deletion | 2 |
| p.Gly106Asp | c.317G>A | Missense | 2 |
| p.Asn166Ser | c.497A>G | Missense | 2 |
| p.Pro205Leu | c.614C>T | Missense | 2 |
| p.Arg213Ser | c.637C>A | Missense | 2 |
| p.Gly254Glu | c.761G>A | Missense | 2 |
| p.Tyr299Cys | c.896A>G | Missense | 2 |
| p.Tyr338Cys | c.1013A>G | Missense | 2 |
|  | c.803-1G>T | Splice acceptor | 2 |
| p.Gln380Ter | c.1138C>T | Nonsense | 2 |
| p.Trp6GlyfsTer111 | c.16del | Frameshift | 1 |
| p.Ser8ArgfsTer109 | c.21del | Frameshift | 1 |
| p.Arg18AlafsTer70 | c.51_61del | Frameshift | 1 |
| p.Arg18ProfsTer99 | c.53del | Frameshift | 1 |
| p.Leu21CysfsTer96 | c.60del | Frameshift | 1 |
| p.Arg32ProfsTer85 | c.95del | Frameshift | 1 |
| p.Ala39ArgfsTer78 | c.115del | Frameshift | 1 |
| p.Arg77ProfsTer40 | c.230del | Frameshift | 1 |
| p.Gly89AlafsTer28 | c.264del | Frameshift | 1 |
| p.Val109CysfsTer8 | c.325del | Frameshift | 1 |
| p.Val173Ter | c.516del | Frameshift | 1 |
| p.Val182TrpfsTer10 | c.544del | Frameshift | 1 |
| p.Thr200GlnfsTer49 | c.598_599del | Frameshift | 1 |
| p.Lys201AsnfsTer46 | c.603_610del | Frameshift | 1 |
| p.Val255SerfsTer16 | c.763del | Frameshift | 1 |
| p.Gln275SerfsTer82 | c.818dup | Frameshift | 1 |
| p.Leu318HisfsTer38 | c.953_954del | Frameshift | 1 |
| p.Met347IlefsTer10 | c.1040dup | Frameshift | 1 |
| p.Arg359GlnfsTer120 | c.1075_1076insA | Frameshift | 1 |
| p.Arg361LeufsTer118 | c.1081_1082insT | Frameshift | 1 |
| p.Gln364AlafsTer17 | c.1090_1135del | Frameshift | 1 |
| p.Pro385HisfsTer11 | c.1154del | Frameshift | 1 |
| p.Pro386HisfsTer10 | c.1155del | Frameshift | 1 |
| p.Gln391ProfsTer72 | c.1172_1190del | Frameshift | 1 |
| p.Ser396AlafsTer73 | c.1186del | Frameshift | 1 |
| p.Ala36_Leu40del | c.106_120del | Inframe deletion | 1 |
| p.Asp45_Leu58del | c.129_170del | Inframe deletion | 1 |
| p.Leu52del | c.155_157del | Inframe deletion | 1 |
| p.Gly106_Thr108del | c.317_325del | Inframe deletion | 1 |
| p.Ala37_Ala39dup | c.109_117dup | Inframe insertion | 1 |
| p.Val85Met | c.253G>A | Missense | 1 |
| p.Gly89Ser | c.265G>A | Missense | 1 |
| p.Asp91Val | c.272A>T | Missense | 1 |
| p.Asp91Glu | c.273C>G | Missense | 1 |
| p.Gly95Ser | c.283G>A | Missense | 1 |
| p.Ala99Thr | c.295G>A | Missense | 1 |
| p.Leu102Arg | c.305T>G | Missense | 1 |
| p.Gly106Arg | c.316G>C | Missense | 1 |
| p.Leu134Pro | c.401T>C | Missense | 1 |
| p.Thr152Pro | c.454A>C | Missense | 1 |
| p.Leu164Pro | c.491T>C | Missense | 1 |
| p.Met189Val | c.565A>G | Missense | 1 |
| p.Met189Thr | c.566T>C | Missense | 1 |
| p.Val191Leu | c.571G>T | Missense | 1 |
| p.Asn192Lys | c.576T>A | Missense | 1 |
| p.Pro205Ser | c.613C>T | Missense | 1 |
| p.Gly212Asp | c.635G>A | Missense | 1 |
| p.Arg213Pro | c.638G>C | Missense | 1 |
| p.Ile214Thr | c.641T>C | Missense | 1 |
| p.Ser219Arg | c.657C>G | Missense | 1 |
| p.Ala231Pro | c.691G>C | Missense | 1 |
| *p.Glu249Lys | c.745G>A | Missense | 1 |
| p.Trp253Ser | c.758G>C | Missense | 1 |
| p.Trp253Cys | c.759G>C | Missense | 1 |
| p.Val257Ile | c.769G>A | Missense | 1 |
| p.Pro262Ala | c.784C>G | Missense | 1 |
| p.Asp298Tyr | c.892G>T | Missense | 1 |
| p.Tyr338Asp | c.1012T>G | Missense | 1 |
| p.Leu358Pro | c.1073T>C | Missense | 1 |
| - | c.266-1G>A | Splice acceptor | 1 |
| - | c.667del | Splice acceptor | 1 |
| - | c.803-2A>G | Splice acceptor | 1 |
| - | c.265+1G>A | Splice donor | 1 |
| - | c.478+2T>C | Splice donor | 1 |
| - | c.664+1G>C | Splice donor | 1 |
| - | c.802+1G>A | Splice donor | 1 |
| - | c.665-7T>G | Splice region | 1 |
| p.Met1? | c.1A>T | Loss of start codon | 1 |
| p.Met1? | c.1A>C | Loss of start codon | 1 |
| p.Trp6Ter | c.18G>A | Nonsense | 1 |
| p.Ser8Ter | c.23C>A | Nonsense | 1 |
| p.Gln22Ter | c.64C>T | Nonsense | 1 |
| p.Trp46Ter | c.138G>A | Nonsense | 1 |
| p.Gln49Ter | c.145C>T | Nonsense | 1 |
| p.Ser269Ter | c.806C>A | Nonsense | 1 |
| p.Gln289Ter | c.865C>T | Nonsense | 1 |
| p.Gln293Ter | c.877C>T | Nonsense | 1 |
| p.Gln377Ter | c.1129C>T | Nonsense | 1 |

* indicates variants of uncertain significant in ClinVar
